# Effect of dietary supplementation of garlic powder and phenyl acetic acid on productive performance, blood haematology, immunity and antioxidant status of broiler chickens

**DOI:** 10.5713/ajas.20.0140

**Published:** 2020-05-12

**Authors:** I. E. Ismail, M. Alagawany, A. E. Taha, N. Puvača, V. Laudadio, V. Tufarelli

**Affiliations:** 1 Poultry Department, Faculty of Agriculture, Zagazig University, Zagazig 44511, Egypt; 2 Department of Animal Husbandry and Animal Wealth Development, Faculty of Veterinary Medicine, Alexandria University, Edfina 22758, Egypt; 3 Department of Engineering Management in Biotechnology, Faculty of Economics and Engineering Management in Novi Sad, University Business Academy in Novi Sad, Cvećarska, Novi Sad, Serbia; 4 Department of DETO, Section of Veterinary Science and Animal Production, University of Bari “Aldo Moro”, Valenzano 70010, Bari, Italy

**Keywords:** Garlic, Phenyl Acetic Acid, Growth, Immunity, Antioxidant, Broiler

## Abstract

**Objective:**

The effect of garlic powder (GP) and phenyl acetic (PA) acid throughout the fattening period of broiler chickens on performance, blood parameters, immune, and antioxidant parameters as well as carcass traits was evaluated.

**Methods:**

A total of 210 day-old Cobb broiler chicks were randomly distributed into seven dietary treatments having five replications with six chicks per replicate. The first group (control) fed a basal diet without supplements, whereas the 2nd, 3rd, and 4th group were fed basal diet plus 0.25, 0.50, and 0.75 g GP/kg diet, respectively and the group 5th, 6th, and 7th were fed on the basal diet plus 0.25, 0.50, and 0.75 g PA/kg diet.

**Results:**

Broiler body weight and gain at 21 and 42 days were increased (p<0.05) with diets supplemented with GP and PA. Red blood cells and hemoglobin were improved in chickens fed diets enriched with GP. Broiler chickens received diets containing either GP or PA recorded the higher values (p<0.05) of total protein, globulin, high-density lipoprotein, immunoglobulin M (IgM), and IgG, superoxide dismutase and total antioxidant capacity; while, blood total cholesterol, low-density lipoprotein, aspartate-aminotransferase, and malondialdehyde were lowered (p<0.05) compared to control-diet. Liver and immune-related organs weight were improved (p<0.05) in broilers fed diet supplemented with GP and PA.

**Conclusion:**

Feeding of GP or PA in diet had positive effects on performance traits and immunological, antioxidant and physiological status of broilers. Thus, the use of tested feed additives as an eco-friendly alternative to antibiotics produced a positive effect on animal health.

## INTRODUCTION

In the recent years broiler production was spread on the large scale in many countries to provide the requirements of animal protein for human. The usage of antibiotics as growth promoters in broiler diet has been forbid because the anxiety of their residues in poultry tissues which lead to the inducement of new strains of microorganism resistant of antibiotic [1]. Poultry meat and egg production, still pain from great losses due to food contamination with harmful bacteria and their influences also on the poultry performance, such as decrease weight and increase of mortality rate [2]. The use of natural feed additives and growth promoters from different sources in animal feeds is an effective way to enhance nutrient utilization and to reduce the antibiotic residues. Phytogenic supplements are plant-derived products used in feeding poultry to maintain performance of livestock species [3]. Among a large number of herbs, garlic has been considered as an effective plant having antioxidant and antimicrobial activities along with multiple beneficial uses [4]. It was reported that phytogenic feed additives improving body weight, feed efficiency ratio, and increased viability of chickens [5]. Garlic (*Allium sativum*) has a lot of beneficial bioactive substances [6]. In a study, Ziarlarimi et al [7] stated that garlic powder (GP) included many organosulfur compounds such as allicin, alliin, ajoene, diallylsulfide, dithiin, and S-allylcysteine, so that garlic as a natural plant-derived feed additives may be successfully used to improve broiler growth. Further, garlic has been widely used as feed additives or growth promoter substance since antiquity [8] and it has been also identified as a medicinal plant in prevention and treatment of many heart, intestinal and metabolic diseases, such as atherosclerosis, thrombosis, dementia, cancer, and diabetes [9]. Several studies reported that garlic has many beneficial properties such as antimicrobial, antioxidative, antithrombotic, antiplatelet aggregator, and antihypertensive in broilers [10,11].

Organic acids or their salts are used as dietary supplement due to their ability on inhibiting microbial growth in nutrients and then maintain the microbial equilibrium in digestive system. Also, they can convert intestinal pH improving consequently feed ingredients solubility, digestion and their absorption [12]. Actually, organic acids celebrated as non-antibiotic growth promoters in poultry nutrition [13]. Acetic acid is one of organic acids which used to inhibit harmful microbial content in gut, modifying pH level and enhancing feed efficiency [14]. Viveros et al [15] reported that organic acids improve digestion and absorption of feed ingredients and modulate positively immunity mucosa layer in broiler chickens. Thus, there is a need to use more effective feeding strategies and economic alternatives for improving health status and performance of poultry.

Therefore, this study was carried out to examine the effects of GP and phenyl acetic (PA) acid on broiler performance, carcass traits and blood haematology, as well as some immune and antioxidant parameters.

## MATERIALS AND METHODS

This study was designed and carried out at the Poultry Department, Faculty of Agriculture, Zagazig University, Zagazig, Egypt. The protocol was approved by the experimentation rules of the Internal Commission for Environmental and Ethics of Zagazig University.

### Experimental design

A total of 210 day-old Cobb broiler chickens were obtained from a commercial hatchery. Chicks were split randomly to seven dietary treatments having five replicates of six birds each. Birds were kept in floor pens supplied with wood shavings. The first group (control) fed a basal diet without supplements, whereas the group 2nd, 3rd, and 4th were fed basal diet plus 0.25, 0.50, and 0.75 g GP/kg diet, respectively; and the group 5th, 6th, and 7th were fed on the basal diet plus 0.25, 0.50, and 0.75 g PA/kg diet. Birds were reared under the same environmental conditions. Room temperature was 33°C at initial experiment, then decreased gradually to reach 25°C at 15 days of age and kept constant. The basal starter diet (1 to 21 days of age) consisted of 23% crude protein (CP) and 3,000 ME kcal/kg, while the finisher diet (22 to 42 days of age) of 20% CP and 3,150 ME kcal/kg, as recommended by NRC [16]. The experimental diets were prepared by blending the basal diet with 0.25, 0.50, and 0.75 g/kg diet of GP or PA acid. The GP was purchased from a local market in Zagazig City (Sharkia, Egypt) and phenylacetic acid (PA; C_8_H_8_O_2_) from El Gomhouria Co., GOMAC (Zagazig, Egypt), then added to diets. Feed and water were offered *ad libitum*.

### Body weight, carcass traits, and blood sampling

Birds of all groups were individually weighed after arriving to the farm. Body weight (BW) and gain were recorded and calculated at 21 and 42 days of age. Birds (four subjects per replicate) were weighed individually and slaughtered after blood samples collection, by neck cut and allowed to bleed. The Bursa of Fabricius, thymus, spleen, liver, heart and empty small intestine were removed and weighed, then calculated the relative weight to live BW. Small intestine length, width and cecum length were also recorded [17].

From the same chickens, blood samples were taken from the wing vein using a sterile syringe into tubes containing ethylenediaminetetraacetic acid to obtain whole blood and plasma to determine blood hematology and biochemical components. Blood heamatology, including red blood cells (RBC) count, hemoglobin (Hb) concentration, packed cell volume (PCV) by standard procedures [18]. Total white blood cells (WBC) count, percentage of heterocytes, percentage of monocytes, and percentage of lymphocytes were analyzed [19].

### Plasma biochemistry and antioxidant status

Blood samples were centrifugated at 3,000 *g*×20 min to obtain the plasma, then stored at −20°C for analysis. The plasma samples were analyzed to determine total protein (TP), albumin (Alb), globulin (Glb), alanine-aminotransferase (ALT) and aspartate-aminotransferase (AST), total cholesterol (TC), triglycerides, high- and low-density lipoprotein (HDL and LDL), superoxide dismutase (SOD), total antioxidant capacity (TAC), malondialdehyde (MDA), immunoglobulins (IgG and IgM) spectrophotometrically using commercial kits.

### Statistical analysis

Data were analyzed using SAS (SAS Software, Inst. Inc., Cary, NC, USA) according to completely randomized design. Differences among groups were separated by Duncan’s multiple range test. The level of significance was pre-set at p<0.05, following the model: Y_ij_ = μ+T_i_+e_ijk_; where: Y_ij_ = individual observation, μ = the overall mean, T_i_ = effect of treatment (i = 1, 2, 3,…7), and e_ijk_ = random error.

## RESULTS

### Growth and carcass traits

The effect of different levels of GP or PA on broiler’s performance as BW and daily gain is shown in Table 1. Broiler BW at 21 and 42 days of age and BW gain during 1 to 21, 21 to 42, and 1 to 42 days of age were significantly (p<0.05) improved in groups fed diet supplemented with GP or PA. Feeding of 0.75 g GP/kg led to heaviest (p<0.05) final BW and gain compared to the other diets.

Relative weight of liver was significantly increased (p< 0.01) when broilers fed different levels of GP and PA compared to control (Table 2). Conversely, post-slaughter weight, heart percentage, and cecum length were not affected by treatments. However, dietary additives recorded the highest values of small intestine length width compared to control (Table 2). In particular, the higher liver relative weight of 2.93% was observed in broilers fed 0.75 g GP/kg diet, whereas the highest small intestine length of 196.35 mm was detected when fed 0.75 g PA/kg compared to the other groups including control-diet.

### Blood hematology and biochemical parameters

The effect of treatments on blood hematology is reported in Table 3. The RBC and Hb were significantly (p<0.01) increased in chicks fed GP, when compared to those treated with 0.50 and 0.75 g/kg of PA and control-diet. Further, chicks fed diet containing 0.75 g GP/kg diet had the highest value of Hb (13.46 g/dL) compared with the other groups. Lymphocytes and heterocytes levels were increased (p<0.05) with GP and PA supplementation than control. However, GP or PA treatments did not elicit a significant alteration in PCV and WBCs among groups (Table 3).

Data in Table 4 show that chickens fed diets containing different levels of either GP or PA (0.25, 0.50, and 0.75 g/kg) had higher (p<0.05) values of TP and Glb than those in control. Also, broilers supplemented with GP or PA had lower (p<0.01) LDL, TC, and AST than control. Conversely, HDL was increased (p<0.01) in all treated groups than control. No significant differences were detected in Alb, triglycerides and ALT levels in birds receiving feed additives.

### Immunity and antioxidant parameters

As reported in Table 5, dietary supplementation of GP and PA increased significantly the broiler immunological parameters (IgM and IgG) and immune organs (relative weight of thymus, Bursa of Fabricius and spleen), in particular when birds fed enriched diets with 0.75 g GP or PA/kg. Compared to control-diet, antioxidant status in terms of SOD and TAC was improved in birds fed GP or PA; where, broiler supplemented with 0.50 and 0.75 g/kg of GP or PA achieved the highest values of SOD and TAC (p<0.05; Table 6). Also, MDA value was significantly (p<0.0021) reduced in groups receiving feed additives (lowest value recorded in 0.75 g GP/kg) compared to control.

## DISCUSSION

Additing of GP and PA in broiler diets enhanced BW and BW gain at 21 and 42 days of age. The obtained results are in agreement with the findings of Khan et al [6] who observed that broiler treated with GP had significantly higher live weight and gain than unsupplemented group. The same trend was also found by Suriya et al [20] who used 0.5% GP in broiler diet. The better effect of garlic as natural feed additives might be due to increased enzymes activity of pancreas, which offer a better environment for digestion and absorption of nutrients. Our results corroborated those of Nourmohammadi and Khosravinia [21] who showed that addition of 30 g citric acid/kg diet significantly improved broiler BW gain. The improvement in BW and gain achieved when broilers fed GP and PA may be due to dialkylpolysulphide as antibacterial compound which in garlic and playing axial role in broiler weight gain [22]. Furthermore, our observations agreed with those obtained by Dibner and Buttin [23] who illustrated that addition of acetic acid to drinking water of chicks almost increased BW and it may be due to improved digestion of protein, energy and retention by reduced microbial competition for nutrients.

In the current study, results showed that supplementing diet with PA or GP improved the relative weight of liver. Similar trend was previously observed with Ashayerizadeh et al [24] who showed that addition of garlic at 1 kg/ton in broiler diet enhanced carcass yield when compared with control. Conversely, Raeesi et al [25] reported that feeding of 0.5%, 1.0%, and 3% garlic decreased only broiler heart weight. On the other hand, Sohail et al [26] postulated that addition of organic acids in chicken diets did not affect carcass traits such as dressing percentage, liver, heart and spleen weights. Small intestine length and average width were higher in broilers received diet supplemented with both feed additives than control group. In parallel, Denli et al [27] observed that intestinal weight and length were significantly affected by administration of a mixture of organic acids (propionic and formic acid salts) in chickens. Moreover, broilers fed diet supplemented with organic acids increased intestinal weight and length at slaughter age of 42 days, enhancing also growth performance and carcass yield [28].

Values of RBC, Hb, lymphocytes and heterocytes in chickens fed either GP- or PA- enriched diets were higher than those in control-diet. In line with our findings, Askar [29] who found that RBCs, Hb and PCV% increased in broiler fed diet supplemented with PA. The same Author observed also an increase of WBCs in PA supplemented groups. In a study, Rehman et al [30] stated that inclusion dietary acetic acid led to an increase only of lymphocytes count and H/L ratio in broiler chickens. Further, Wang et al [31] when supplemented broiler diet with PA found an increase in WBC and lymphocyte being in agreement with our findings. It was reported by Haque et al [32] that the lymphoid organs content of lymphocytes improved with addition citric acid in diet due to enhanced non-specific immunity. Our results are partially in agreement with previous findings observing that Hb and PCV were not affected by dietary GP in broilers [12]. Also, Elagib et al [33] found that no effect of diets containing 3% and 5% GP on broiler RBC, PCV, and WBC. The hemolytic bioactive and their metabolites in garlic may be responsible of these effects. The improvement in RBC with GP supplementation could be due to the end-product produced from garlic metabolism possible stimulates formation and secretion of erythropoietin of the kidney directly to cause erythrocyte synthesis [34]. Dorhoi et al [35] demonstrated that garlic extract addition to laying hens diet tended to enhance spleen RBCs uptake.

Findings of our study showed that supplementing diets with GP and PA decreased blood TC and LDL, and increased TP, Glb, and HDL. These results are partially in agreement with Rahimi et al [36] who found in broilers that blood triglycerides, TC, and LDL concentrations were decreased, while HDL increased when fed enriched-diet with 0.1% GP. At this regard, Issa and Omar [37] showed that addition of GP at 0.2% and 0.4% reduced significantly concentrations of triglycerides, cholesterol, LDL in Cobb broiler, increasing the level of HDL compared to control. On the contrary, it was reported no differences in serum biochemical parameters (TP, Alb, Glb, cholesterol, triglycerides, ALT, and AST) of broilers fed diet enriched with garlic [6]. The potential effect of garlic products may be due to depressing the lipogenic and cholesterogenic activity of liver enzymes such as fatty acid synthase, glucose-6-phosphatase dehydrogenase, malic enzyme, and 3-hydroxy-3-methylglutaryl-CoA reductase, consequently, the mechanism of hypocholesterol and hypolipid syntheses [38].

In a study, Rehman et al [30] reported that broilers fed diet supplemented with acetic acid had the highest value of TP and Glb comparing to control diet. Previously, Askar [29] observed that birds receiving 0.075%, 0.150%, and 0.225% PA had lower value of cholesterol than control group. The increase in Glb led to improved immunity, and Rahmani and Speer [39] demonstrated that dietary inclusion of organic acid increased concentration of serum Glb and induced better immune response in broilers. Our dietary treatments decreased ALT levels in comparison to control, and this result partially agreed those of Askar [29] who found that addition of PA reduced significantly ALT than control. The same trend was obtained by Salgado-Tránsito et al [40] who found that 50 g/kg citric acid in broiler diet decreased ALT activity; however, increased serum AST concentration. Further, Nourmohammadi and Khosravinia [21] showed that citric acid supplemented into diet at 60 g/kg significantly reduced concentrations of HDL and AST than control birds.

A significant increase was observed in the broiler serum IgG and IgM and weights of thymus, Bursa of Fabricius and spleen when fed GP and PA. These improvements in immunological parameters may be due to the bioactive molecules in GP and PA. In agreement with our results, Rahimi et al [36] found that dietary supplementation of garlic improved the relative weight of Bursa of Fabricius than non-treated birds. Conversely, Raeesi et al [25] reported that chicks fed diet with 0.5%, 1.0%, and 3% garlic had lower weights of bursa and spleen. The same trend of our study was also found by Hossain et al [41] who reported that *L. mesenteroides*-fermented garlic did not affect Bursa Fabricius. Hanieh et al [28] observed that dietary garlic supplementation increased IgG concentration. In the same trend, Askar [29] showed that relative weight of Bursa of Fabricius and spleen significantly increased in broiler treated with PA, whereas, Sohail et al [26] reported that organic acids addition did not affect spleen weight of broilers.

In the GP- and PA-treated groups, the levels of TAC and SOD were significantly increased at 0.75 g/kg; while, the highest concentration of MDA was recorded in control group. Our results agreed those obtained by Pourali et al [42] who indicated that adding garlic to broiler diet decreased of MDA concentrations by 30% compared to birds fed basal-diet, without affecting SOD. Recently, Nasiroleslami et al [43] showed that broiler fed diet supplemented with guanidinoacetic acid significantly improved glutathione peroxidase activity in broiler liver and decreased serum MDA. Furthermore, Alagawany et al [44] demonstrated that dietary supplementation of garlic in diet had positive impact on SOD and TAC activities.

In conclusion, based on our findings, both GP and PA acid, as natural feed additives in diet, had antioxidant and immune effects in broilers, having also influence on their growth performance and blood parameters and lipid peroxidation. In particular, both natural feed additives resulted more effective when supplemented at the rate of 0.50 or 0.75 g/kg diet.

## Figures and Tables

**Table 1:** Live body weight and body weight gain of broiler chicks as affected by dietary garlic powder and phenyl acetic acid supplementation

Treatment	Live BW (g/bird)		Daily BW gain (g/d/bird)		
	
	21 days	42 days	1–21 days	21–42 days	1–42 days
Control	928.1^c^	1,986.3^d^	41.64^c^	50.38^d^	46.93^c^
GP (g/kg)					
0.25	945.5^c^	2,035.2^c^	42.26^c^	51.91^d^	47.53^c^
0.50	1,033.3^a^	2,175.2^b^	47.11^a^	54.18^b^	52.62^b^
0.75	1,052.7^a^	2,245.1^a^	47.85^a^	56.81^a^	55.42^a^
PA (g/kg)					
0.25	985.6^b^	2,159.6^b^	45.85^b^	55.32^b^	48.21^c^
0.50	993.9^b^	2,190.4^b^	45.37^b^	57.21^a^	51.28^a^
0.75	943.6^c^	2,065.1^c^	42.14^c^	53.42^c^	47.74^c^
SEM	18.75	23.01	8.173	1.513	4.074
Pr>f	0.0124	0.0087	0.0279	0.0193	0.00126

BW, body weight; GP, garlic powder; PA, phenyl acetic; SEM, standard error of the mean.

a–d Means within the same column with different common superscripts differ.

**Table 2:** Carcass traits of broiler chicks as affected by dietary garlic powder and phenyl acetic acid supplementation

Treatment	Post-slaughter weight (g)	Liver (%)	Heart (%)	Cecum length (cm)	Small intestine measurements		

					Width (mm)	Length (mm)	Weight (g)
Control	1,876.6	1.61^c^	0.456	14.67	8.67^b^	154.67^c^	114.34
GP (g/kg)							
0.25	1,954.1	2.36^ab^	0.597	17.96	10.61^a^	183.99^b^	135.01
0.50	1,943.3	2.57^ab^	0.603	19.21	11.21^a^	150.34^c^	122.35
0.75	1,871.5	2.93^a^	0.671	21.63	10.64^a^	180.67^b^	115.43
PA (g/kg)							
0.25	1,859.6	2.18^b^	0.572	20.43	7.85^b^	179.35^b^	114.12
0.50	1,828.3	2.64^ab^	0.569	17.82	8.13^b^	150.41^c^	130.67
0.75	1,885.1	2.81^a^	0.655	17.37	10.42^a^	196.35^a^	122.36
SEM	48.57	0.668	0.086	1.523	4.018	5.493	8.175
Pr>f	0.1870	0.0017	0.0680	0.1007	0.0004	0.0001	0.0583

GP, garlic powder; PA, phenyl acetic; SEM, standard error of the mean.

a–c Means within the same column with different common superscripts differ.

**Table 3:** Blood hematology of broiler chicks as affected by dietary garlic powder and phenyl acetic acid supplementation

Treatment	RBC (×10^6^/mL)	PCV (%)	Hb (g/dL)	WBC (×10^3^/mL)	Monocytes (%)	Lymphocytes (%)	Heterophils (%)
Control	2.84^ab^	38.03	9.27^c^	22.27	4.87	33.08^b^	42.13^d^
GP (g/kg)							
0.25	2.96^ab^	40.66	11.93^ab^	23.89	5.46	45.23^a^	51.38^bc^
0.50	3.28^a^	38.84	12.71^ab^	24.18	5.31	47.51^a^	57.68^ab^
0.75	3.16^a^	42.22	13.46^a^	26.21	4.97	52.42^a^	59.62^ab^
PA (g/kg)							
0.25	3.01^ab^	43.95	11.34^ab^	23.44	5.73	48.13^a^	62.28^a^
0.50	2.89^bc^	39.01	10.52^bc^	21.97	4.81	47.32^a^	47.01^cd^
0.75	2.61^bc^	38.93	10.11^bc^	23.65	5.62	46.78^a^	58.53^ab^
SEM	1.328	4.007	1.881	3.075	2.609	4.915	4.063
Pr>f	0.0081	0.1290	0.0100	0.1800	0.084	0.0490	0.0015

RBC, red blood cells; PCV, packed cell volume; Hb, hemoglobin; WBC, red blood cells; GP, garlic powder; PA, phenyl acetic; SEM, standard error of the mean.

a–d Means within the same column with different common superscripts differ.

**Table 4:** Blood biochemistry parameters of broiler chicks as affected by dietary garlic powder and phenyl acetic acid supplementation

Treatment	TP (g/dL)	Alb (g/dL)	Glb (g/dL)	TC (mg/dL)	HDL (mg/dL)	LDL (mg/dL)	Triglycerides (g/dL)	AST (μ/L)	ALT (μ/L)
Control	3.01^b^	1.78	1.23^b^	95.64^a^	17.84^c^	42.41^a^	72.76	246.81^a^	12.63
GP (g/kg)									
0.25	4.06^b^	2.55	1.51^ab^	72.51^bc^	26.67^bc^	26.71^b^	58.37	196.63^b^	9.96
0.50	4.51^ab^	2.58	1.93^a^	64.75^bc^	28.79^ab^	24.23^b^	50.18	170.55^bc^	7.82
0.75	4.87^a^	2.76	2.11^a^	58.87^c^	38.24^a^	21.97^b^	48.71	138.94^c^	6.91
PA (g/kg)									
0.25	4.22^b^	2.31	1.91^a^	71.63^bc^	21.44^bc^	30.76^b^	55.42	186.88^c^	8.24
0.50	4.21^b^	2.49	1.72^a^	63.21^bc^	21.36^bc^	27.65^b^	61.78	201.07^b^	10.63
0.75	3.96^b^	2.19	1.77^a^	81.17^ab^	27.04^bc^	24.91^b^	58.21	172.34^bc^	9.98
SEM	1.549	0.546	0.164	14.758	5.589	13.954	21.134	38.619	3.765
Pr>f	0.002	0.198	0.029	0.006	0.008	0.004	0.149	0.005	0.221

TP, total protein; Alb, albumin; Glb, globulin; TC, total cholesterol; HDL, high-density lipoprotein; LDL, low-density lipoprotein; AST, aspartate-aminotransferase; ALT, alanine-aminotransferase; GP, garlic powder; PA, phenyl acetic; SEM, standard error of the mean.

a–c Means within the same column with different common superscripts differ.

**Table 5:** Immune parameters of broiler chicks as affected by dietary garlic powder and phenyl acetic acid supplementation

Treatment	Relative weight of immune organs			IgM (mg/dL)	IgG (mg/dL)

	Bursa Fabricius (%)	Thymus (%)	Spleen (%)		
Control	0.092^b^	0.395^b^	0.068^c^	71.81^d^	195.32^b^
GP (g/kg)					
0.25	0.161^ab^	0.578^ab^	0.121^ab^	100.67^cd^	295.65^ab^
0.50	0.167^a^	0.719^a^	0.128^ab^	112.92^bc^	298.87^ab^
0.75	0.183^a^	0.753^a^	0.151^a^	149.89^a^	335.85^a^
PA (g/kg)					
0.25	0.162^ab^	0.548^ab^	0.112^b^	96.68^cd^	317.35^ab^
0.50	0.173^a^	0.681^a^	0.127^ab^	111.92^bc^	328.95^ab^
0.75	0.163^ab^	0.756^a^	0.137^ab^	130.87^ab^	357.44^a^
SEM	0.0362	0.1308	0.0134	20.161	154.112
Pr>f	0.0367	0.0134	0.0095	0.0010	0.0310

Ig, immunoglobulin; GP, garlic powder; PA, phenyl acetic; SEM, standard error of the mean.

a–d Means within the same column with different common superscripts differ.

**Table 6:** Antioxidant status of broiler chicks as affected by dietary garlic powder and phenyl acetic acid supplementation

Treatment	SOD (nmol/mL)	TAC (nmol/mL)	MDA (nmol/mL)
Control	0.036^c^	0.116^d^	0.285^a^
GP (g/kg)			
0.25	0.181abc	0.212abc	0.158^b^
0.50	0.189^ab^	0.231abc	0.104^b^
0.75	0.254^a^	0.281^a^	0.098^b^
PA (g/kg)			
0.25	0.131^bc^	0.163^cd^	0165^b^
0.50	0.147^bc^	0.206^bc^	0.161^b^
0.75	0.224^ab^	0.247^ab^	0.146^b^
SEM	0.053	0.054	0.070
Pr>f	0.0240	0.0027	0.0021

SOD, superoxide dismutase; TAC, total antioxidant capacity; MDA, malondialdehyde; GP, garlic powder; PA, phenyl acetic; SEM, standard error of the mean.

a–d Means within the same column with different common superscripts differ (p<0.05) or (p<0.01).
